# Supplementary material for: Molecular basis of human nuclear and mitochondrial tRNA 3′ processing
Source: Nat Struct Mol Biol. 2025 Jan 2;32(4):613–24. doi: 10.1038/s41594-024-01445-w (PMC11996679; doi:10.1038/s41594-024-01445-w)

## Figure 1d

**Region shown in Figure 1d is indicated by dashed box**

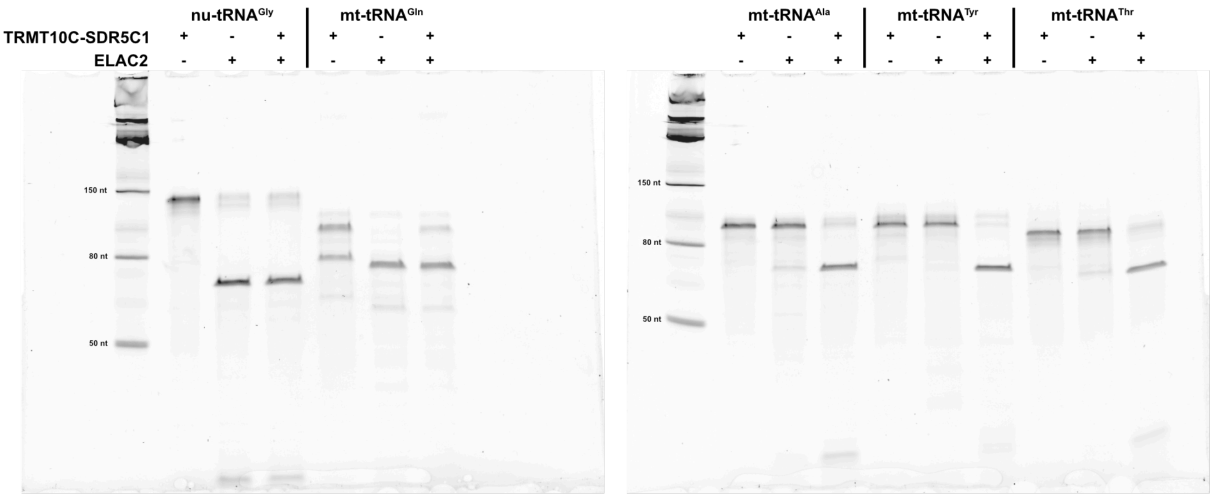


Replicate 1

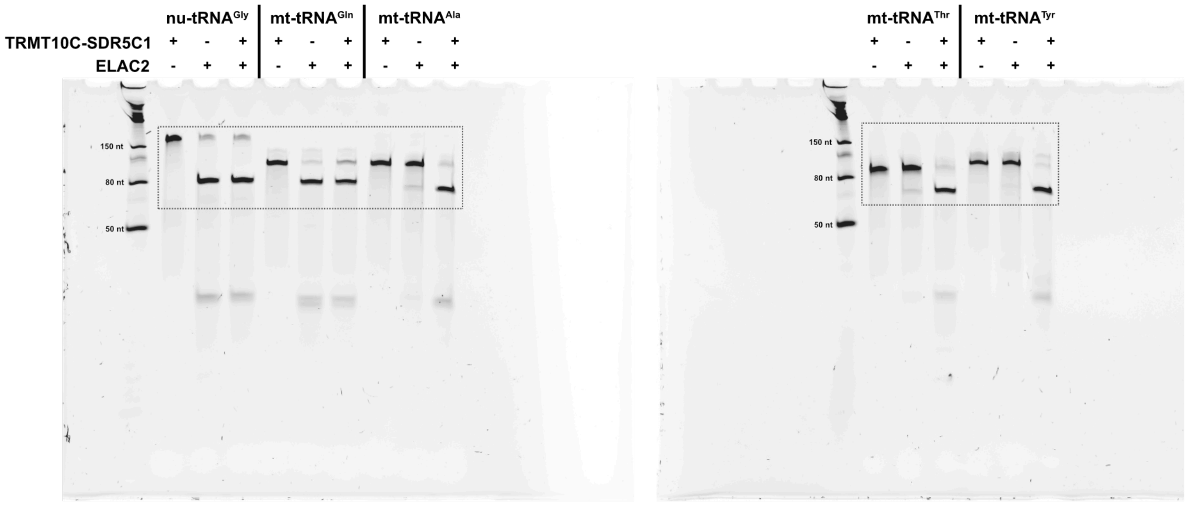


Replicate 2

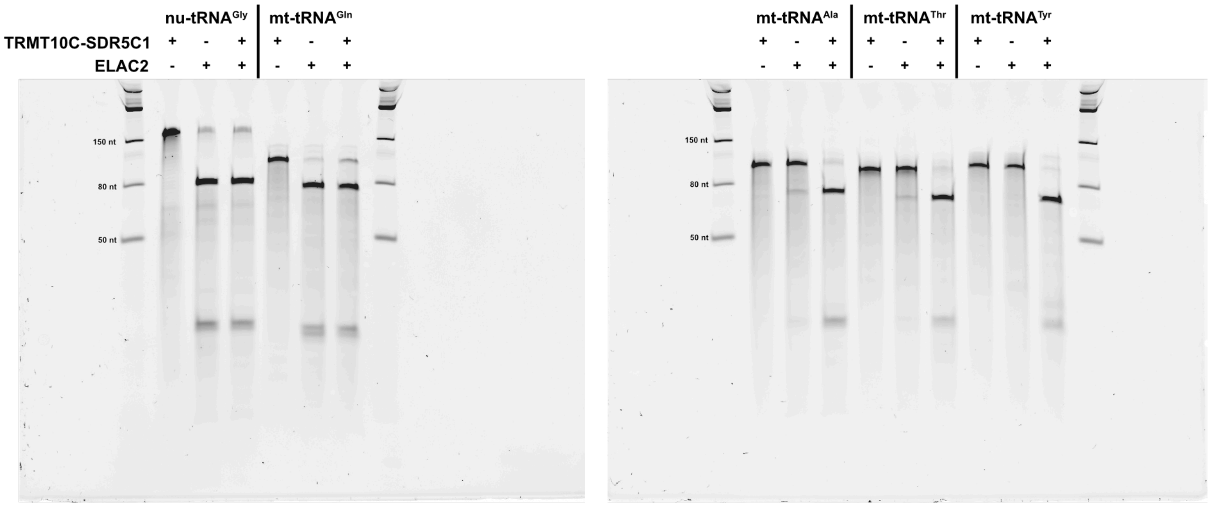


Replicate 3

# Figure 1e

**Region shown in Figure 1e is indicated by dashed box**

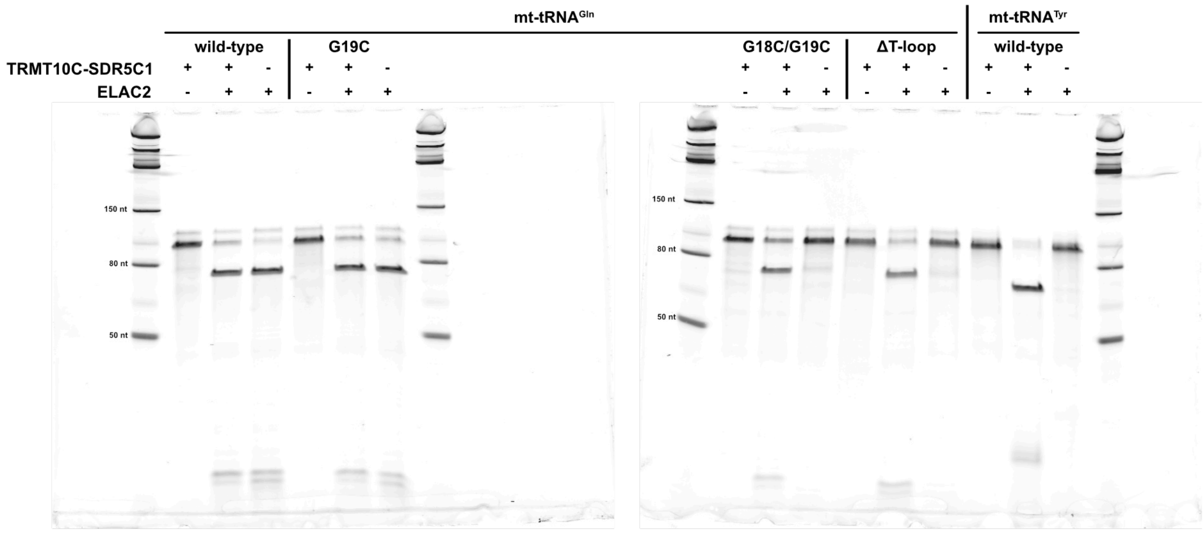


Replicate 1

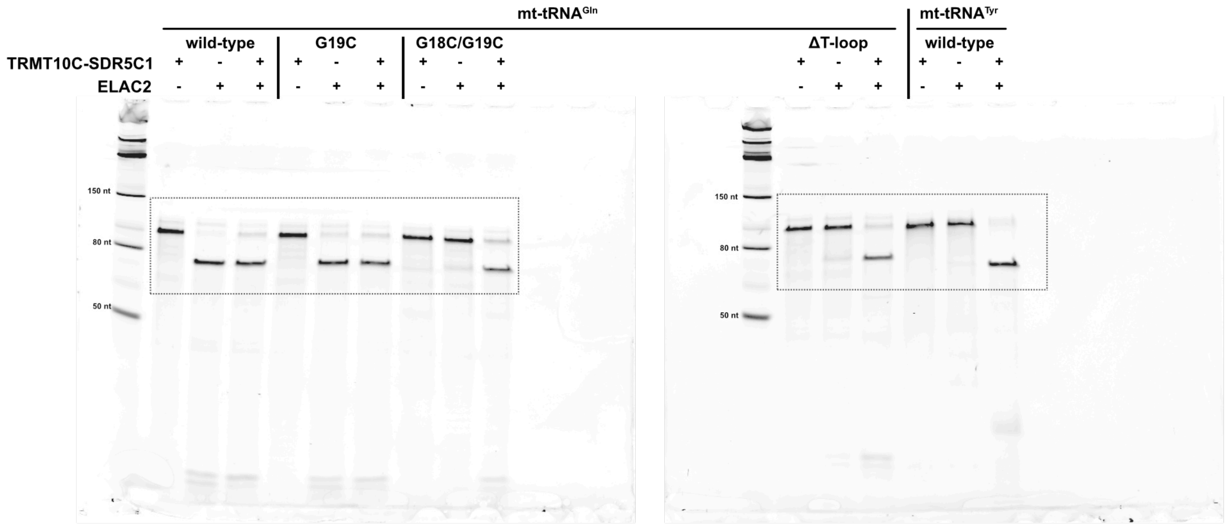


Replicate 2

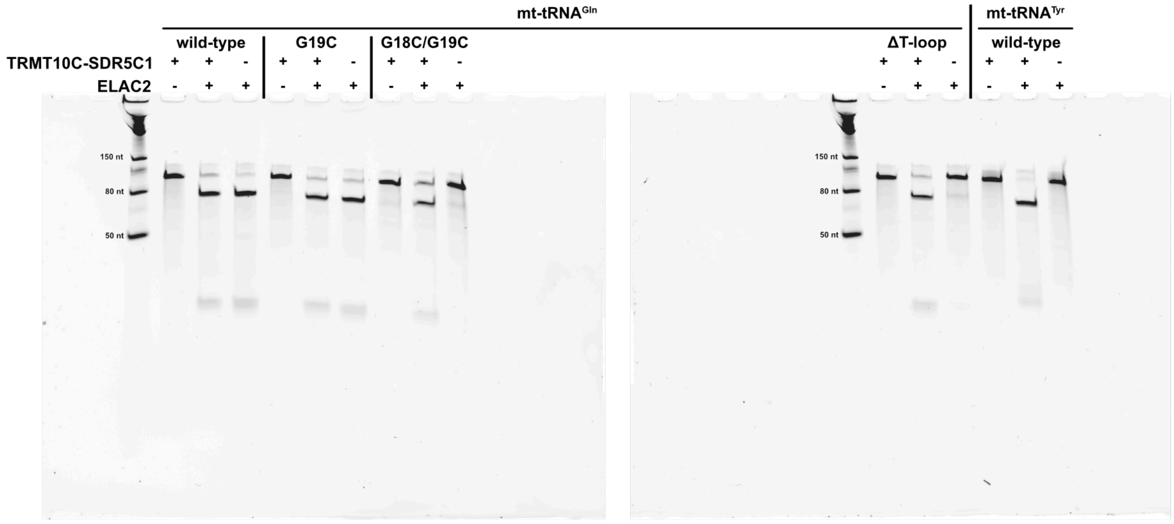


Replicate 3

# Figure 3g and 5g

**Regions shown in Figures 3g and 5g are indicated by dashed boxes**

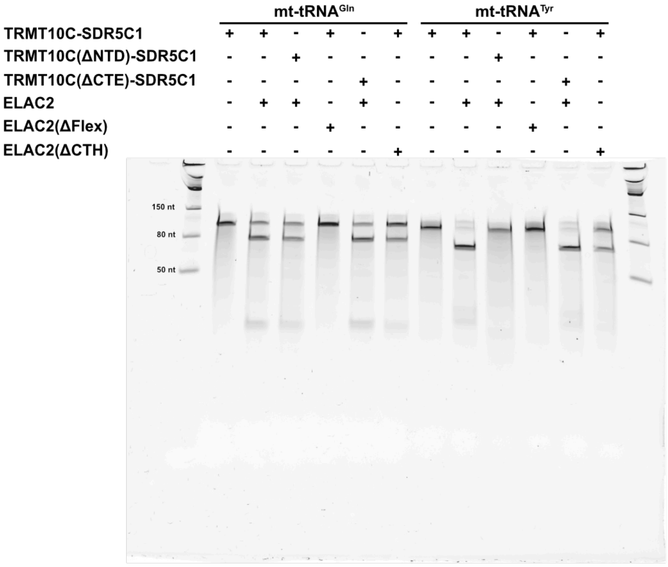


Replicate 1

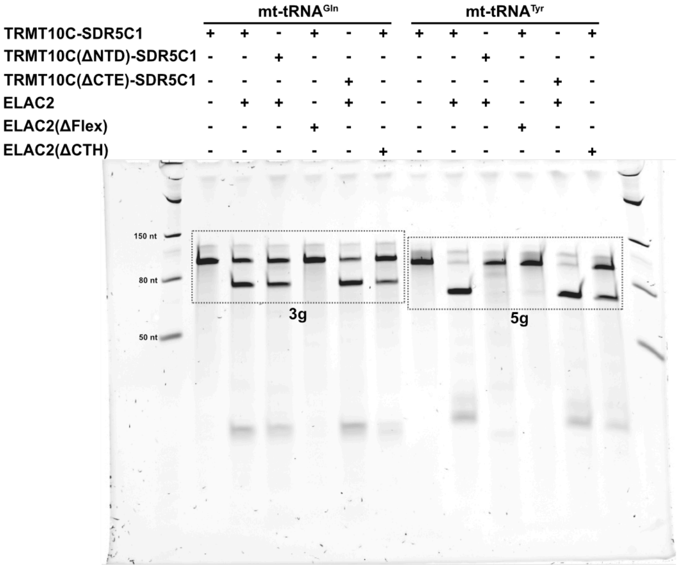


Replicate 2

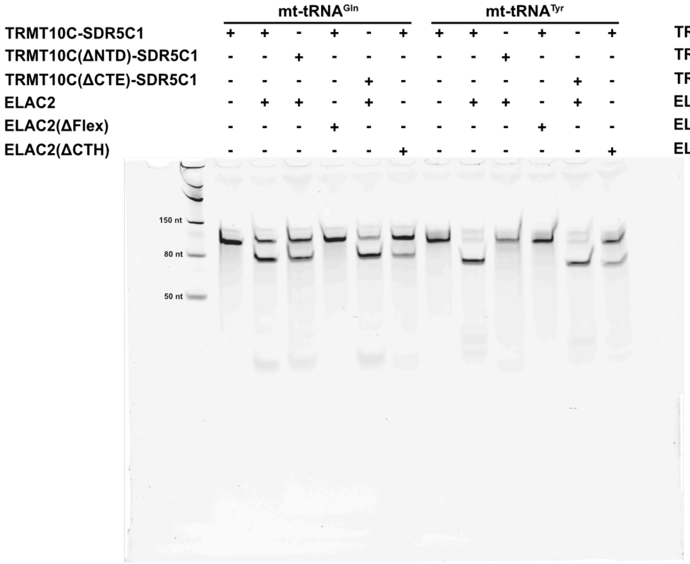


Replicate 3

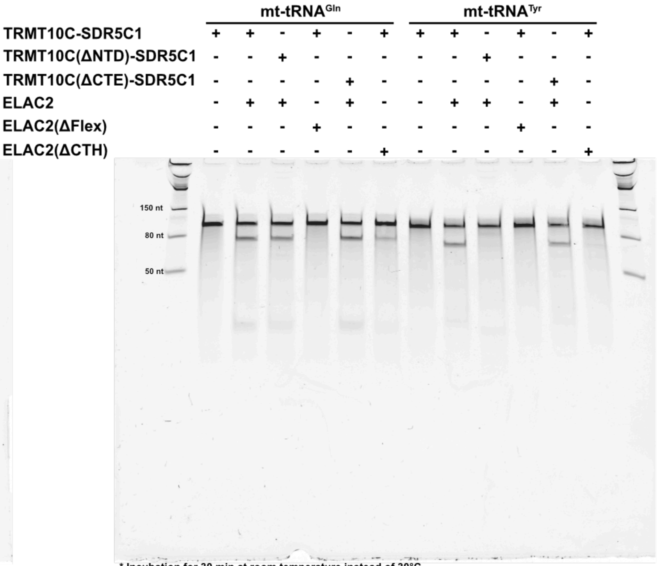


* Incubation for 30 min at room temperature instead of 30°C

Replicate 4

## Figure 4c

**Region shown in Figure 4c is indicated by dashed boxes**

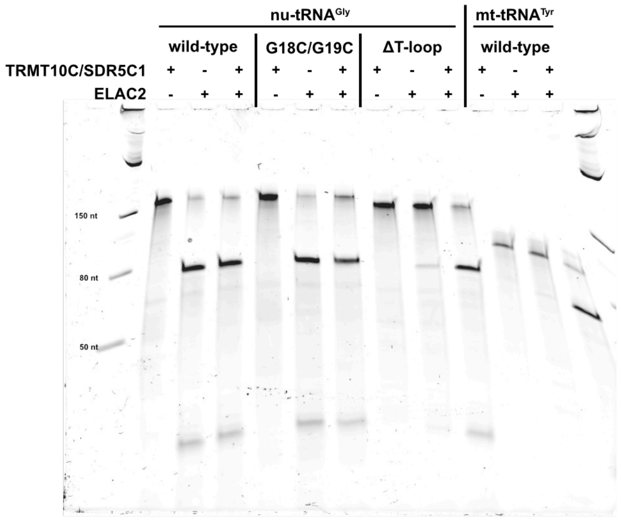


Replicate 1

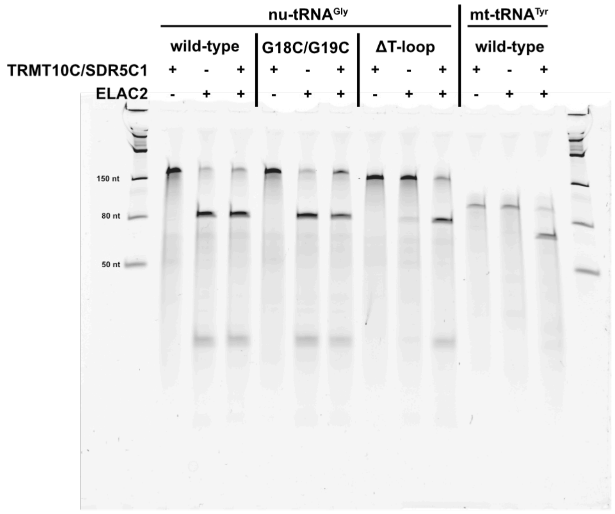


Replicate 2

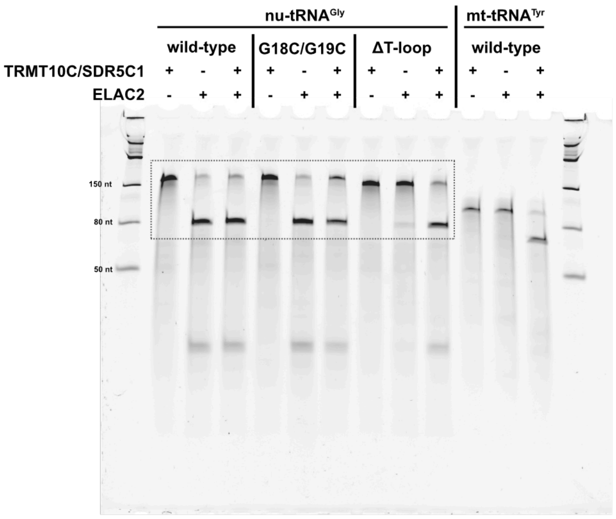


Replicate 3

## Extended Data Figure 1b

**Region shown in ED-Figure 1b is indicated by dashed box**

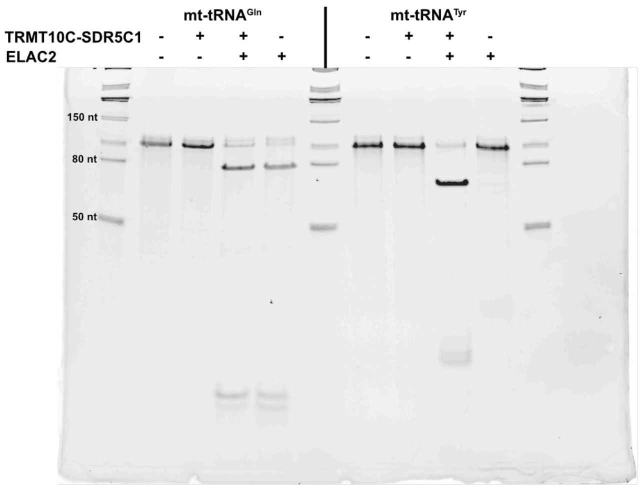


Replicate 1

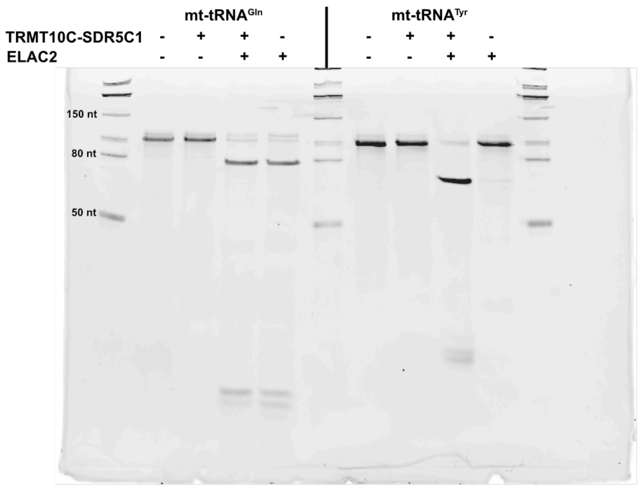


Replicate 2

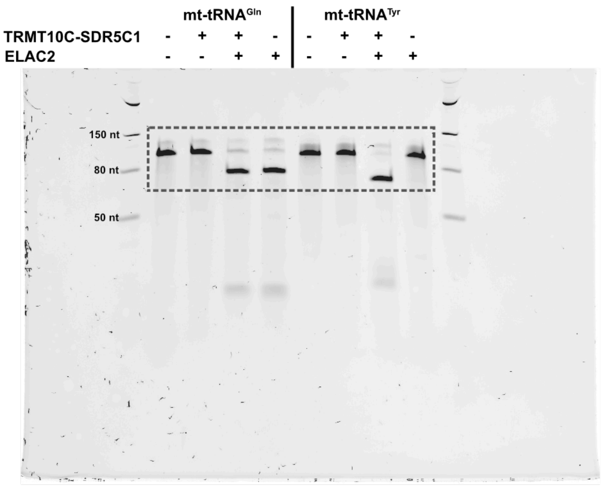


Replicate 3

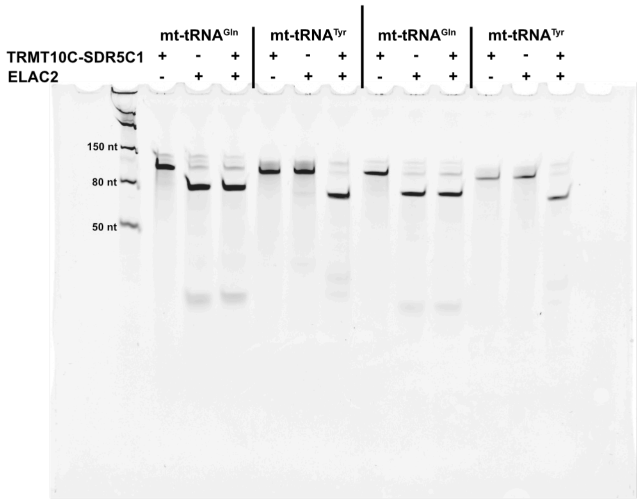


Replicate 4

## Extended Data Figure 1d

**Regions shown in ED-Figure 1d is indicated by dashed box**

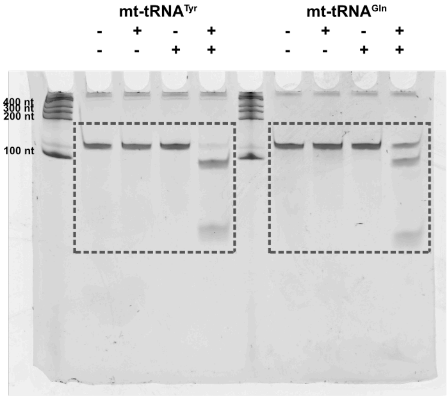


Replicate 1

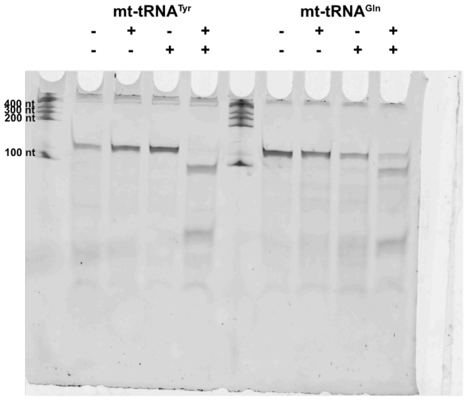


Replicate 2

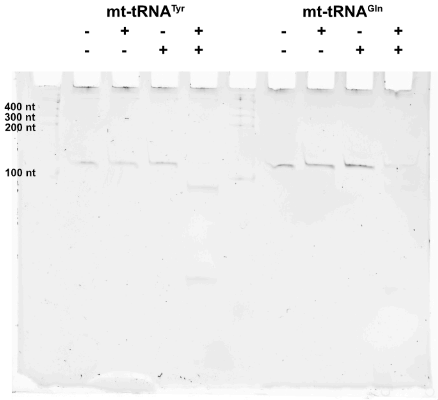


Replicate 3

## Extended Data Figure 2a

**Region shown in ED-Figure 2a is indicated by dashed box**

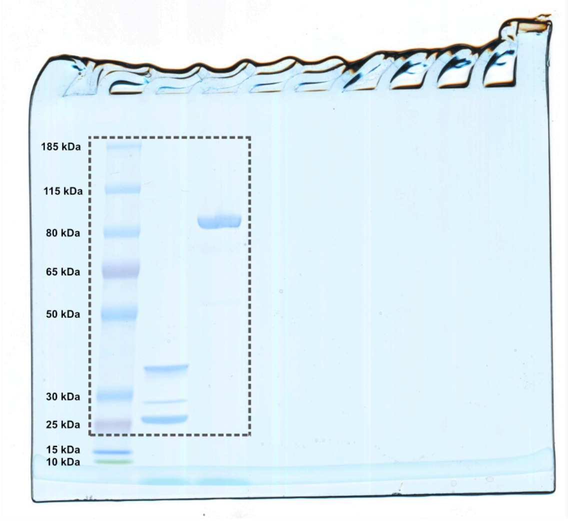


## Extended Data Figure 2b

**Regions shown in ED-Figure 2b are indicated by dashed boxes**

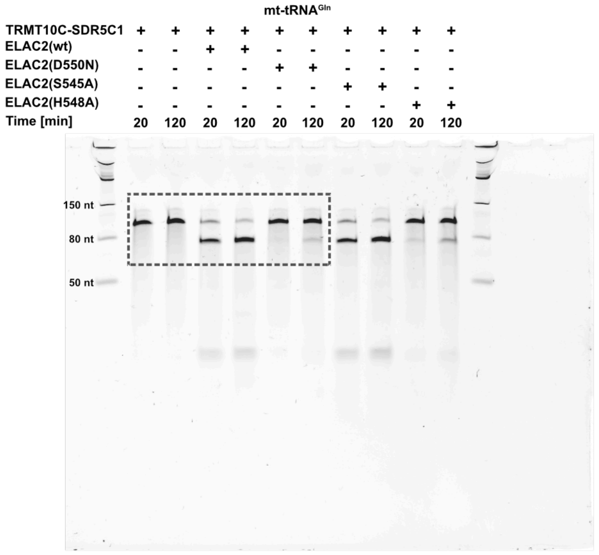


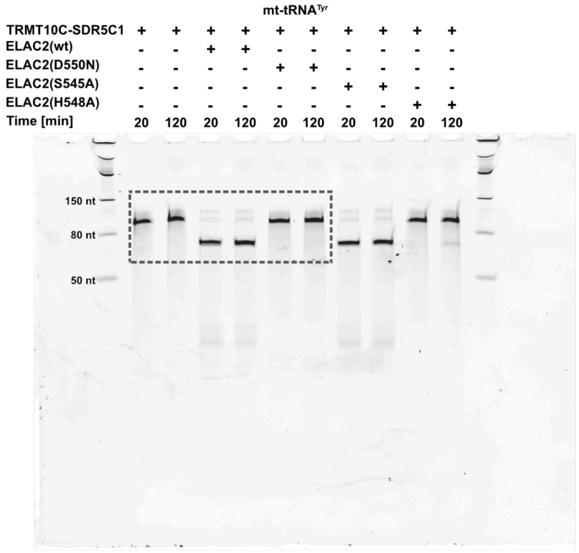


## Extended Data Figure 2c

**Region shown in ED-Figure 2c is indicated by dashed boxes**

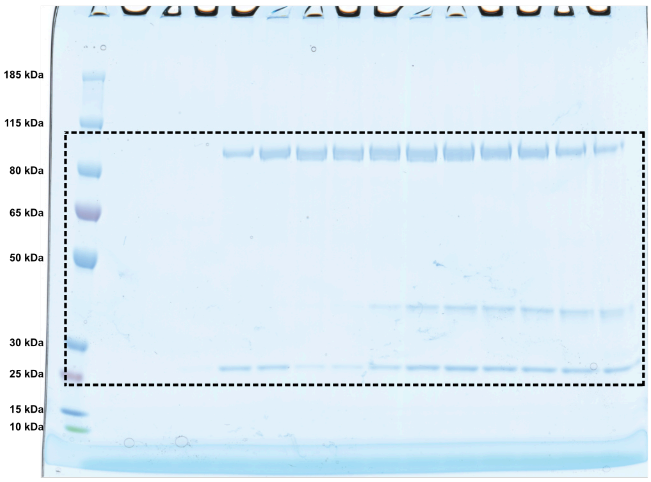


## Extended Data Figure 2d

**Region shown in ED-Figure 2d is indicated by dashed boxes**

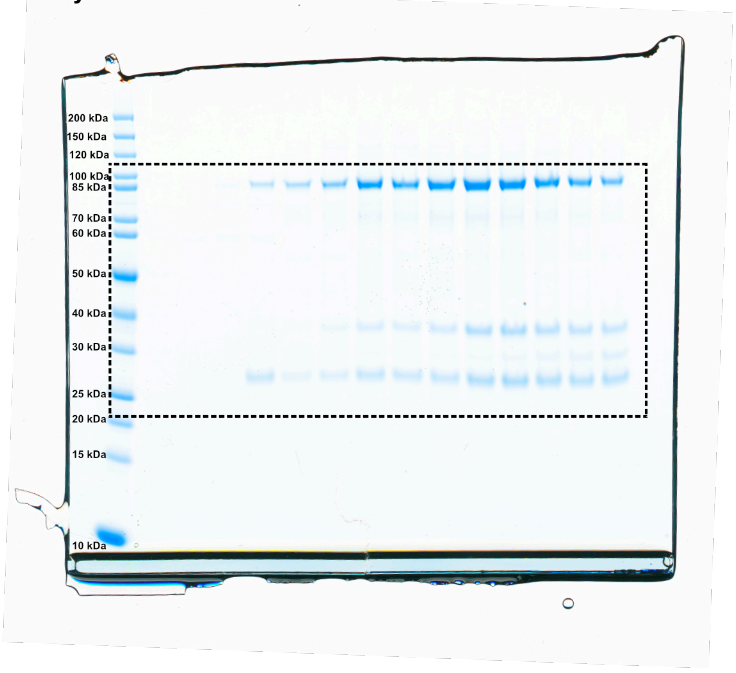

## Extended Data Figure 7f

**Regions shown in ED-Figure 7f are indicated by dashed boxes**

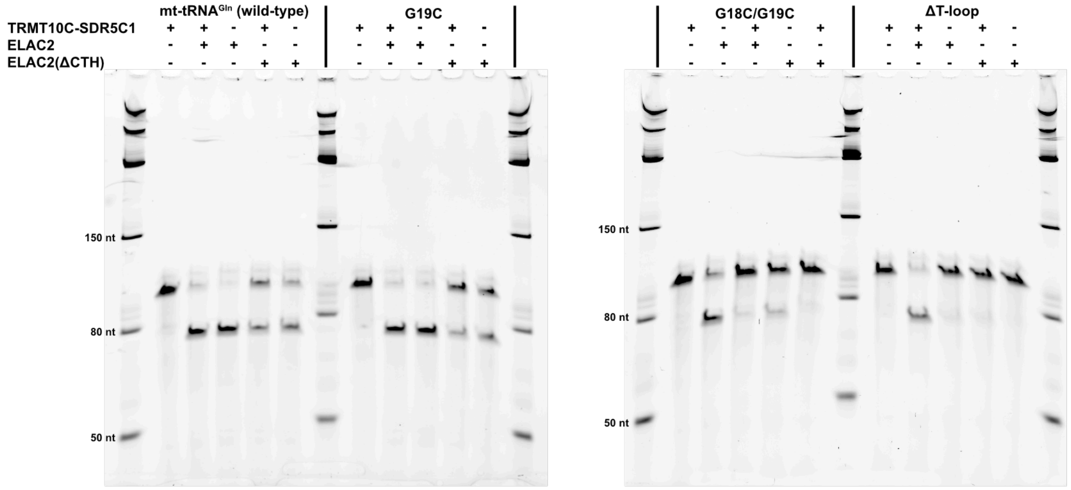


Replicate 1

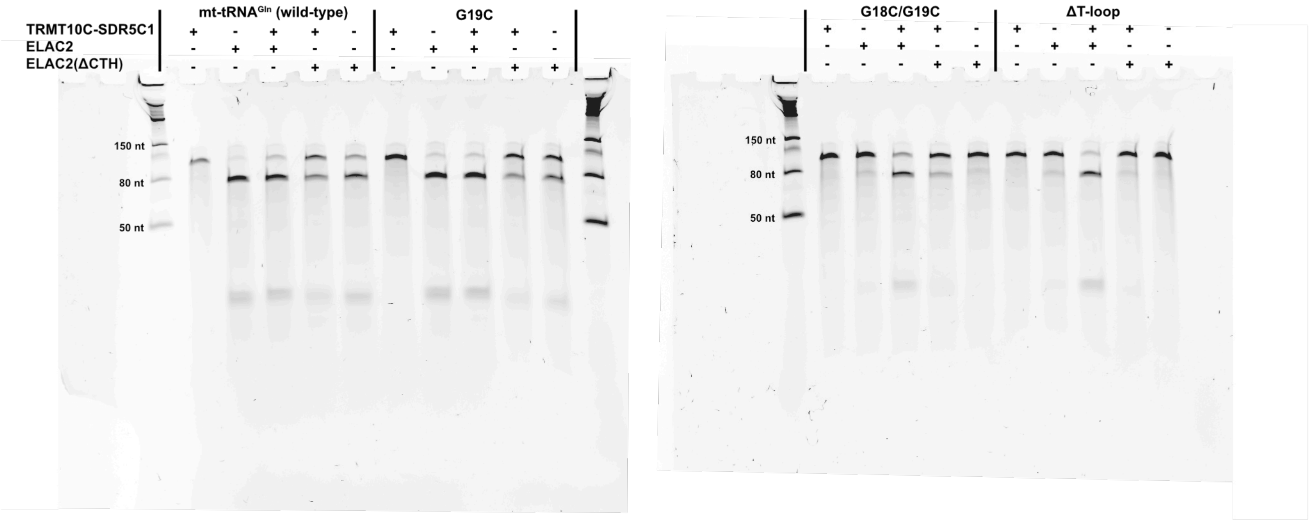


Replicate 2

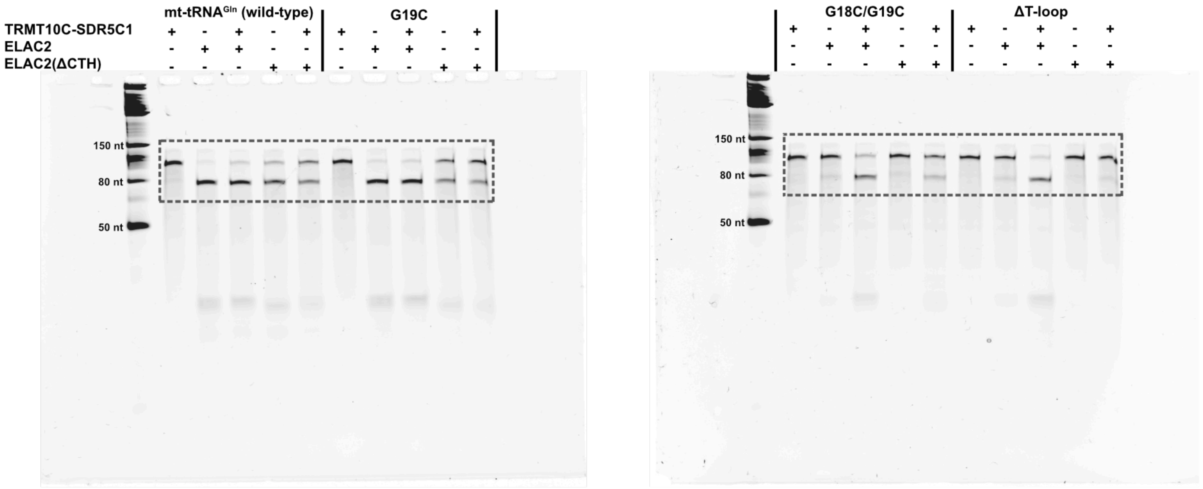


Replicate 3

# Extended Data Figure 9e

## Region shown in ED-Figure 9e is indicated by dashed box

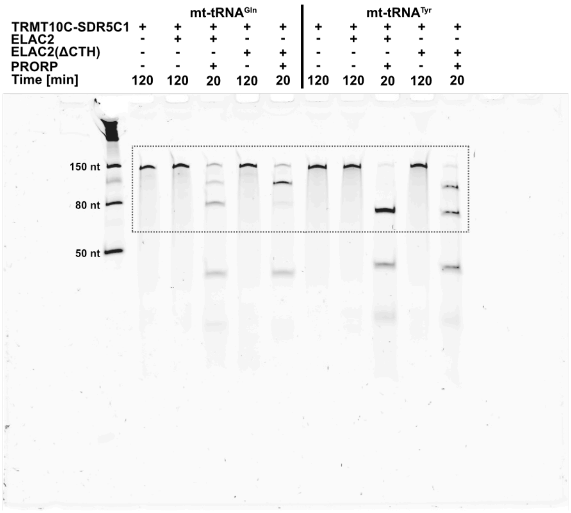

Supplement: Supplementary file 4 — Images of unprocessed SDS–PAGE and urea PAGE gels for Figs. 1d,e, 3g, 4c and 5g and Extended Data Figs. 1b,d, 2a–d, 7f and 9e. [file 41594_2024_1445_MOESM4_ESM.pdf]
